# EFL Students’ L2 Achievement: The Role of Teachers’ Organizational Commitment and Loving Pedagogy

**DOI:** 10.3389/fpsyg.2022.937624

**Published:** 2022-07-06

**Authors:** Di Ye, Suping Sun, Dongmei Zhao

**Affiliations:** School of Foreign Studies, Tangshan Normal University, Tangshan, China

**Keywords:** teacher organizational commitment, L2 achievement, loving pedagogy, EFL students, EFL

## Abstract

Since student L2 achievement is the primary objective of all language education contexts, a huge number of inquiries have explored the role of students’ personal characteristics in promoting their L2 achievement. A plethora of studies have also assessed the impact of teachers’ personal and professional qualities on students’ L2 achievement. Yet, the effects of teachers’ organizational commitment and loving pedagogy have largely been neglected. In addition, no review article has been undertaken to outline the consequences of teachers’ organizational commitment and loving pedagogy for students’ L2 achievement. Against this backdrop, the present article intends to describe the role of teachers’ organizational commitment and loving pedagogy in EFL students’ L2 achievement by reviewing the theoretical and empirical evidence. The review revealed that teachers’ organizational commitment and loving pedagogy can favorably influence EFL students’ L2 achievement. The implications for language teachers, teacher trainers, and educational administrators are also discussed.

## Introduction

The academic mastery of a second/foreign language, which is called L2 achievement, is the sole objective of any language education context. Simply put, helping learners to attain higher L2 achievement is what language education contexts, including EFL contexts, are designed for. Student achievement, according to Gajda et al. (2017), pertains to “the outcome of learning, which is typically measured by classroom grades, classroom assessments, and external achievement tests” (p. 271). In a similar vein, student L2 achievement refers to “the overall attainment of students in a language course” (Wang S. et al., 2022, p. 1). It actually pertains to the amount of linguistic knowledge accumulated by language learners throughout a whole course (Ma, 2022). In a nutshell, L2 achievement is concerned with how far language learners have been successful in mastering the language learning materials. With respect to the fact that students’ L2 achievement is subjective to their personal qualities (Othman and Leng, 2011; Feng et al., 2013), a multitude of inquiries have been conducted on the role of students’ personal qualities in their L2 achievement (e.g., Erten and Burden, 2014; Soodmand Afshar et al., 2014; Li and Lynch, 2016; Subekti, 2018; Shih, 2019; Taheri et al., 2019; Li, 2020; Li and Wei, 2022, among others). Additionally, owing to the fact that teachers as the main source of knowledge can substantially influence their students’ academic competence and performance (Akbari and Allvar, 2010; Kola and Sunday, 2015; Kim and Seo, 2018), a plethora of investigations have explored the impact of teachers’ personal and professional qualities on students’ L2 achievement (e.g., Mojavezi and Tamiz, 2012; Gowrie and Ramdass, 2014; Rashidi and Moghadam, 2014; Baghaei and Riasati, 2015; Mahmoodi et al., 2015; Klusmann et al., 2016; Kheirzadeh and Sistani, 2018; Ma et al., 2018, among others). Notwithstanding, the implications of teachers’ organizational commitment and loving pedagogy for EFL students’ L2 achievement have been largely disregarded by researchers. Simply put, little attention has been devoted to the consequences of these professional qualities (i.e., organizational commitment, loving pedagogy) for students’ L2 achievement (Aliakbari and Amoli, 2016; Altun, 2017; Lu, 2021).

The concept of organizational commitment, also known as professional commitment, broadly refers to the depth and strength of an employee’s affiliation to an organization (Allen and Meyer, 1990). According to Greenberg and Baron (2008), organizational commitment pertains to “the extent to which an individual identifies and is involved with his or her organization and/or is unwilling to leave it” (p. 234). Likewise, teacher organizational commitment refers to the degree to which an individual teacher is involved in and identifies with a certain educational setting (Zhu et al., 2011). As Ibrahim and Iqbal (2015) noted, those teachers who deeply identify themselves with their universities, schools, or institutes typically do their best for the success and progress of these educational organizations. Dong and Xu (2022) also stated that teachers that are strongly committed to their profession continuously endeavor to lead their pupils toward increased achievement. It implies that the greater the teachers’ professional commitment, the higher the students’ achievement.

The construct of loving pedagogy reflects the extent to which “teachers are sensitive and caring in their perspectives, as well as understanding and respectful of their students” (Yin et al., 2019, p. 2). As Loreman (2011) noted, love is demonstrated in the educational contexts through a supportive atmosphere, close bond between the instructor and learners, and classroom practices. A loving teacher is believed to be capable of improving his/her students’ academic achievement, motivation, wellbeing, and engagement (Wang et al., 2021; Zhao and Li, 2021). As Zhao and Li (2021) mentioned, through a loving pedagogical approach, teachers can encourage learners to put their all into fulfilling their academic responsibilities. In this respect, Wang et al. (2021) posited that loving pedagogy enables instructors to involve students in all phases of learning. Active involvement in the learning process will result in increased student achievement (Lei et al., 2018; Tao et al., 2022).

Notwithstanding the value of teachers’ organizational commitment and loving pedagogy in improving students’ academic achievement (Zhao and Li, 2021; Dong and Xu, 2022), the consequences of these variables for students’ achievement have been rarely explored. More specifically, the role of these constructs in increasing students’ L2 achievement has remained elusive. That is, limited investigations have been done in this respect (Aliakbari and Amoli, 2016; Altun, 2017; Lu, 2021). To make a stride toward addressing this gap, this review article seeks to clearly explain the role of teachers’ organizational commitment and loving pedagogy in EFL students’ L2 achievement.

### Organizational Commitment

The notion of organizational commitment generally refers to “the relative strength of an individual’s identification with and involvement in a particular organization” (George and Sabapathy, 2011, p. 92). In this respect, teacher organizational commitment pertains to individual teachers’ identification, alignment, and engagement with an educational institution (Ni, 2017). According to Fathi and Savadi Rostami (2018), teacher organizational commitment has something to do with teachers’ positive attitudes toward a given educational organization that links their identity to that particular organization. As a complex construct, teacher organizational commitment comprises three components of *“affective commitment,” “continuous commitment,”* and *“normative commitment”* (McShane and Von Glinow, 2010). The first component, affective commitment, relates to the emotional attachment and psychological connection an individual teacher has with the educational institution and its academic purposes (Huang and You, 2011). The second component, continuous commitment, involves teachers’ willingness to remain in an educational institution due to the close bond they have with their learners, colleagues, and the subject matter they teach (Rusu, 2013). Normative commitment, on the other hand, refers to the teachers’ willingness to continue working for an educational institution due to moral and ethical issues (Sow et al., 2016).

### Loving Pedagogy

The notion of *“loving pedagogy”* or *“pedagogy of love”* pertains to “the care, sensitivity, and empathy that teachers have toward their students’ needs, learning experiences, and development” (Zhao and Li, 2021, p. 2). The concept of loving pedagogy, according to Loreman (2011), encompasses nine emotional notions, including kindness, bonding, empathy, sacrifice, intimacy, passion, acceptance, forgiveness, and community. A loving pedagogy is a combination of these nine notions between the instructors and learners (Page, 2018). According to Luguetti et al. (2019), instructing learners through love inspires them to ardently pursue the long, arduous journey of learning. Wang Y. et al. (2022) have also modified the previous models of “loving pedagogy,” adding the traditional Chinese education philosophy and cultural norms, and they also stressed the value of love in education through referring to its positive influences on students’ engagement, academic growth, and learning outcomes.

### The Role of Teachers’ Organizational Commitment and Loving Pedagogy in EFL Students’ L2 Achievement

The positive role of teachers’ organizational commitment in students’ L2 achievement can be explained through what Fathi and Savadi Rostami (2018) articulated in this regard. They stated that committed teachers tend to create favorable changes in their learners’ academic outcomes as they are emotionally and psychologically attached to them (Lee et al., 2011). In this respect, Dong and Xu (2022) posited that individual teachers’ sense of commitment leads them to devote substantial endeavors to educating their learners. In light of the fact that devoting adequate time and effort to educating students is a prerequisite for enhancing their learning achievement (Park, 2005), they argued that dedicated teachers will be more successful in improving their students’ achievement as they give their all in educating their students. Besides, the favorable role of loving pedagogy in students’ L2 achievement can be justified in light of positive psychology (PP) principles which underline the value of positive emotions (e.g., joy, happiness, love, etc.) in education. Building upon PP principles, Wilkinson and Kaukko (2020) maintained that educating students through love will significantly contribute to their increased achievement. They asserted that adopting a loving pedagogical approach helps teachers establish a mutual bond with their students that is crucial for improving their engagement, motivation, and achievement.

## Empirical Evidence

As mentioned earlier in this study, the role of teachers’ organizational commitment and loving pedagogy in increasing student achievement has been kept under the carpet. Simply put, the impacts of these variables on student achievement have largely been ignored by researchers. It means that a few researchers have studied the potential consequences of teachers’ organizational commitment and loving pedagogy for student achievement (Aliakbari and Amoli, 2016; Altun, 2017; Lu, 2021). For one, Aliakbari and Amoli (2016) evaluated the associations between teacher commitment and student L2 achievement. In doing so, the reliable measures of teacher commitment and student achievement were given to 356 EFL students and teachers. Assessing the correlations of the scales, they discovered a direct relationship between teacher commitment and student L2 achievement. In another inquiry, Altun (2017) explored the influence of teacher organizational commitment on student achievement. To accomplish this, 100 students were invited to take part in interview sessions. The findings of the interviews revealed that teachers’ organizational commitment can promote student achievement. In a similar vein, Lu (2021) assessed the role of teacher commitment in EFL students’ academic success. To do so, a group of Chinese EFL students were asked to answer the related questionnaires. The findings indicated that teachers’ commitment can positively contribute to EFL students’ academic success. Having reviewed the related literature, we can find that the previous studies on EFL teachers’ organizational commitment and loving pedagogy mainly focus on the quantitative research methods or conceptualization. It is worth noting that, the validated questionnaires about loving pedagogy are still lacking. Emotion is a complex multidimensional construct, so it is recommended to do some longitudinal or indepth narrative studies.

## Conclusion and Pedagogical Implications

Previously in this review article, the constructs of teacher organizational commitment, loving pedagogy, and student L2 achievement along with their components were characterized. Furthermore, through theoretical and empirical basis, the role of teacher organizational commitment and loving pedagogy in EFL students’ L2 achievement was outlined. Considering the theoretical and empirical basis, it is fair to conclude that teacher organizational commitment and loving pedagogy can make a noticeable difference in EFL students’ L2 achievement. Simply said, EFL students’ L2 achievement is subjective to the teachers’ level of commitment and teaching method. This seems to be insightful for all second and foreign language teachers, teacher trainers, and educational administrators. It is deemed illuminating for all language teachers in that they can understand the prominence of organizational commitment and loving pedagogy in promoting students’ language achievement. In doing so, teachers and students will build up a harmonious atmosphere, which will help them to actively engage in the class activities. For instance, in the authentic teaching practice, language teachers may be encountered with some struggling students who have great difficulty in learning. In particular, in my own education context of China, there are some students who come from a minority community, for example, Qinghai, Yunnan, Xinjiang or Tibet provinces. Those students have been exposed to multilingual context. That is to say, they need to study two other non-mother tongue languages, Chinese and English besides their own language. Towards these special group of students, teachers need to show more love, more care, more patience to help those students to actively engage in the language learning. Then students can have a confident feeling and mood to study and speak English. Then in these minority students’ thoughts, they want to study English, and really love English. With teachers’ help and some changes of their own, we believe that students’ English level can gradually improve. The finding of this review is also perceived enlightening for teacher trainers in that they can educate teachers on how to teach students through love. They can also brief them on how to remain committed to their profession, workplace, and learners. Additionally, with regard to the fact that working conditions can remarkably affect one’s organizational commitment, educational administrators are expected to provide teachers with an appropriate working environment to enhance their organizational commitment.

## Author Contributions

All authors have made a direct and intellectual contribution to the work and got it ready for its publication.

## Conflict of Interest

The authors declare that the research was conducted in the absence of any commercial or financial relationships that could be construed as a potential conflict of interest.

## Publisher’s Note

All claims expressed in this article are solely those of the authors and do not necessarily represent those of their affiliated organizations, or those of the publisher, the editors and the reviewers. Any product that may be evaluated in this article, or claim that may be made by its manufacturer, is not guaranteed or endorsed by the publisher.
